# Clinical outcomes with second-line dolutegravir in people with virological failure on first-line non-nucleoside reverse transcriptase inhibitor-based regimens in South Africa: a retrospective cohort study

**DOI:** 10.1016/S2214-109X(23)00516-8

**Published:** 2023-12-21

**Authors:** Kwabena Asare, Yukteshwar Sookrajh, Johan van der Molen, Thokozani Khubone, Lara Lewis, Richard J Lessells, Kogieleum Naidoo, Phelelani Sosibo, Rosemary van Heerden, Nigel Garrett, Jienchi Dorward

**Affiliations:** a Centre for the AIDS Programme of Research in South Africa (CAPRISA), Durban, South Africa; b eThekwini Municipality Health Unit, eThekwini Municipality, Durban, South Africa; c KwaZulu-Natal Research and Innovation Sequencing Platform (KRISP), University of KwaZulu-Natal, Durban, South Africa; d Discipline of Public Health Medicine, School of Nursing and Public Health, University of KwaZulu-Natal, Durban, South Africa; e South African Medical Research Council (SAMRC)-CAPRISA-TB-HIV Pathogenesis and Treatment Research Unit, Nelson R Mandela School of Medicine, University of KwaZulu-Natal, Durban, South Africa; f Nuffield Department of Primary Care Health Sciences, University of Oxford, Oxford, UK

## Abstract

**Background:**

Dolutegravir (DTG) is recommended for second-line antiretroviral therapy (ART) after virological failure on first-line non-nucleoside reverse transcriptase inhibitor (NNRTI)-based regimens in people living with HIV in low-income and middle-income countries. We compared the effectiveness of DTG versus the previously recommended ritonavir-boosted lopinavir (LPV/r) regimen for second-line treatment in South Africa.

**Methods:**

In this retrospective observational cohort study, we used routinely collected, de-identified data from 59 primary health-care facilities in eThekwini Municipality, KwaZulu-Natal, South Africa. We included people living with HIV aged 15 years or older with virological failure (defined as two consecutive viral loads of ≥1000 copies per mL at least 56 days apart) on first-line NNRTI-based ART containing tenofovir disoproxil fumarate (TDF) and who switched to second-line ART. Our primary outcomes were retention in care and viral suppression (<50 copies per mL) at 12 months after starting second-line treatment. We used modified Poisson regression models to compare these outcomes between second-line regimens (zidovudine [AZT]/emtricitabine or lamivudine [XTC]/DTG; TDF/XTC/DTG; and AZT/XTC/LPV/r).

**Findings:**

We included 1214 participants in our study, of whom 729 (60%) were female and 485 (40%) were male, and whose median age was 36 years (IQR 30–42). 689 (57%) were switched to AZT/XTC/LPV/r, 217 (18%) to AZT/XTC/DTG, and 308 (25%) to TDF/XTC/DTG. Compared with AZT/XTC/LPV/r (75%), retention in care was higher with AZT/XTC/DTG (86%, adjusted risk ratio [aRR]=1·14, 95% CI 1·03–1·27; adjusted risk difference [aRD]=10·89%, 95% CI 2·01 to 19·78) but similar with TDF/XTC/DTG (77%, aRR=1·01, 0·94–1·10; aRD=1·04%, –5·03 to 7·12). Observed retention in care was lower with TDF/XTC/DTG than with AZT/XTC/DTG, although in multivariable analysis evidence for a difference was weak (aRR=0·89, 0·78–1·01, p=0·060; aRD=–9·85%, –20·33 to 0·63, p=0·066). Of 799 participants who were retained in care with a 12-month viral load test done, viral suppression was higher with AZT/XTC/DTG (59%; aRR=1·25, 1·06–1·47; aRD=11·57%, 2·37 to 20·76) and higher with TDF/XTC/DTG (61%; aRR=1·30, 1·14–1·48; aRD=14·16%, 7·14 to 21·18) than with AZT/XTC/LPV/r (47%).

**Interpretation:**

These findings from routine care support further implementation of WHO's recommendation to use DTG instead of LPV/r in people living with HIV who experience virological failure while receiving first-line NNRTI-based ART.

**Funding:**

Bill & Melinda Gates Foundation.

**Translation:**

For the isiZulu translation of the abstract see Supplementary Materials section.

## Introduction

Following WHO recommendations,^1^, ^2^ dolutegravir (DTG) has been implemented for second-line antiretroviral therapy (ART) in people with HIV with virological failure on first-line non-nucleoside reverse transcriptase inhibitor (NNRTI)-based regimens in South Africa since December, 2019, replacing previously recommended lopinavir–ritonavir (LPV/r)-based regimens.^3^, ^4^ The WHO recommendations were based on results from the DAWNING trial^5^ showing superior efficacy of DTG for second-line ART compared with LPV/r. Afterwards, evidence from the NADIA trial^6^ showed that recycling first-line tenofovir disoproxil fumarate (TDF) in a DTG-based second-line ART was non-inferior to switching to zidovudine (AZT). However, there are little data from routine care demonstrating the effectiveness of DTG, either with AZT or recycling TDF, on clinical outcomes during second-line ART.

Before December 2019, people living with HIV in South Africa who were receiving the standard first-line regimen of TDF, emtricitabine (FTC) and efavirenz (EFV), and presented with virological failure (repeat viral load ≥1000 copies per mL 2–3 months apart), were recommended to switch to a second-line regimen of zidovudine (AZT), lamivudine (3TC), and LPV/r.^4^ After DTG was introduced for second-line ART in 2019, they were recommended to switch to AZT/3TC/DTG. Some people with virological failure during first-line treatment might have been switched to TDF/3TC/DTG, either inadvertently as part of the transition to first-line DTG or by clinicians following preliminary evidence suggesting that TDF/3TC/DTG might be an effective second-line regimen.^7^ As the rollout of DTG in low-income and middle-income countries (LMICs) continues, evidence on the effectiveness of different regimens in routine care settings is required to guide further rollout and confirm clinical trial findings.^7^, ^8^

**Research in context**

**Evidence before this study**

We searched PubMed from inception until May 30, 2023, with no language restrictions, for published articles evaluating outcomes with dolutegravir (DTG)–zidovudine (AZT)-based regimens versus DTG–tenofovir disoproxil fumarate (TDF)-based regimens versus ritonavir-boosted lopinavir (LPV/r)-based regimens for second-line antiretroviral therapy. We used the search terms [dolutegravir AND (tenofovir OR lopinavir-ritonavir) AND (second-line antiretroviral therapy)]. We found five clinical trials (DAWNING, NADIA, D2EFT, VISEND, and ARTIST) and zero observational studies. The DAWNING trial showed the superiority of DTG versus LPV/r when used with two nucleoside reverse transcriptase inhibitors (NRTIs) in 624 participants who had had previous first-line treatment failure (≥400 copies per mL) with non-nucleoside reverse transcriptase inhibitor (NNRTI)-based regimens. At week 48 after baseline, 261 (84%) of 312 participants in the DTG group had viral suppression (<50 copies per mL) compared with 219 (70%) of 312 in the LPV/r group. Among 464 participants in the NADIA trial who had had first-line treatment failure (≥1000 copies per mL) on an NNRTI with TDF and either lamivudine or emtricitabine (XTC), recycled TDF for second-line treatment was non-inferior at week 48 compared with AZT (90·2% *vs* 91·7%), all used with DTG or darunavir for viral suppression (<400 copies per mL). The VISEND and D2EFT trials demonstrated the non-inferiority of DTG with TDF and XTC to standard-of-care ritonavir-boosted protease inhibitors lopinavir, atazanavir, and darunavir for second-line treatment. In the single-arm ARTIST trial, including 62 participants with virological failure on first-line TDF and XTC with efavirenz (EFV) or nevirapine (NVP) and switched to second-line regimens with recycled TDF and DTG, viral suppression (<50 copies per mL) was 74·0% at 48 weeks. These clinical trials, except the ARTIST trial, have demonstrated the effectiveness of second-line DTG used with AZT or recycled first-line TDF for viral suppression compared with previous standard-of-care ritonavir-boosted protease inhibitor-based regimens. However, outcomes in non-trial or routine health-care settings, where treatment adherence might be relatively lower than in trial settings, are scarce. Furthermore, the relative effectiveness of these second-line regimens for retention in care, probably due to regimen tolerability within an antiretroviral treatment programme setting, is also limited.

**Added value of this study**

Since the implementation of DTG for second-line antiretroviral treatment in low-income and middle-income countries, this is, to the best of our knowledge, the first study using ART programme data from routine health-care clinics to assess outcomes of 12-month retention in care and viral suppression after switching to second-line DTG used with AZT or recycled first-line TDF versus the previously recommended LPV/r. DTG was better when used with AZT but similar when used with recycled TDF for retention in care, and all treatments were better for viral suppression than the previous LPV/r. The effect of DTG was lower for retention in care when used with recycled TDF than when used with AZT, although evidence of a difference was weak, and similar for viral suppression.

**Implications of all the available evidence**

Evidence from ongoing real-world cohorts through ART programmatic data evaluation is important for confirming the usefulness of common regimen combinations in regular health-care settings to guide further decision-making. We have provided evidence outside clinical trial settings that supports WHO's recommendation of DTG use replacing LPV/r for second-line treatment in resource-limited settings. Our findings also suggest that recycling first-line TDF instead of replacing it with AZT for a DTG-based second-line regimen can be an effective alternative for viral suppression. Further evidence from routine care settings on adverse events and resistance mutations during second-line DTG-based treatment would be a vital addition to evidence for continuous improvement of ART guidelines.

Therefore, we aimed to assess the effectiveness of DTG plus either FTC or 3TC (XTC) in combination with AZT or TDF versus the previously recommended AZT/XTC/LPV/r regimen for second-line treatment in people who experienced virological failure while taking an NNRTI-based first-line ART in routine health-care clinics in South Africa.

## Methods

### Study design and participants

We did a retrospective observational cohort study with de-identified, routinely collected data from South Africa's ART programme in 59 of approximately 100 public sector primary health-care clinics in the eThekwini Municipality of the KwaZulu-Natal province. The study was approved by the Biomedical Research Ethics Committee of the University of Kwazulu-Natal (BE646/17), the KwaZulu-Natal Provincial Health Research Ethics Committee (KZ_201807_021), the TB/HIV Information Systems Data Request Committee, and the eThekwini Municipality Health Unit.

Our study population included all people living with HIV aged 15 years or older who were switched to a second-line ART between Dec 1, 2019, and Nov 30, 2020. We used this baseline period of switching to second-line therapy to allow a minimum follow-up duration of 12 months plus 90 days before the data cutoff on April 21, 2022. We included only people who were previously receiving standard first-line regimens of TDF/XTC/EFV or TDF/XTC/NVP at the time of virological failure (defined as two consecutive viral loads ≥1000 copies per mL at least 56 days apart) and were switched to second-line regimens of AZT/XTC/LPV/r, AZT/XTC/DTG, and TDF/XTC/DTG. Thus, we excluded people who were switched to a four-drug regimen of AZT/3TC/TDF plus LPV/r or DTG (ie, participants co-infected with hepatitis B) and those switched to abacavir-based second-line regimens.

### Procedures

South Africa's ART delivery in public health-care clinics involves clinical assessment for pregnancy, viral load, CD4 count testing, and screening for tuberculosis at baseline ART initiation and follow-up visits. Based on the South African 2019 ART treatment guidelines,^4^ which were in use at the time of this study (from Dec 1, 2019, to April 21, 2022), viral load was routinely taken at 6 and 12 months after ART initiation and every 12 months thereafter, unless viraemia occurred. CD4 count was measured at ART initiation and 12 months thereafter and then only repeated if clinically indicated (eg, viral load ≥1000 copies per mL). People living with HIV with a viral load of 1000 copies per mL or higher were recommended to receive enhanced adherence counselling and a repeat viral load testing after 2–3 months. For people receiving first-line regimens containing an NNRTI such as efavirenz [EFV] or nevirapine [NVP], virological failure was defined as two consecutive viral loads of 1000 copies per mL or higher 2–3 months apart. If virological failure occurred, switching to second-line ART was recommended. There was no routine testing for HIV drug resistance at the time of first-line ART virological failure in this setting.

### Data sources and data management

We used data from South Africa's TIER.Net electronic database, which contains demographics, clinical status, regimen, and clinic visit information of people receiving ART in public sector health-care clinics.^9^ Data on intermediate outcomes such as the provision of adherence counselling and adverse events were unavailable in the TIER.Net electronic database and hence could not be analysed in this study. Data were de-identified by the South African National Department of Health's TB/HIV Information Systems before access and analysis by the study team.

### Outcomes and exposures

Our primary outcomes were retention in care and viral suppression at 12 months after starting second-line treatment. Retention in care at 12 months was defined as not being lost to follow-up or recorded in TIER.Net as either deceased or transferred out to another clinic (as we could not access or link to data at other clinics to establish retention in care) by 365 days after starting second-line treatment. We defined loss to follow-up using the South African ART programme guidelines as being 90 days late for a scheduled visit.^10^ Viral suppression was defined as viral load lower than 50 copies per mL. We included one secondary outcome for a post-hoc sensitivity analysis defining viral suppression as viral load lower than 1000 copies per mL. Because viral loads are not always completed regularly in routine care, we defined the 12-month window as the closest viral load to 365 days between 181 and 545 days after starting second-line treatment and included only the viral loads of participants retained in care.

The primary exposure was the second-line ART regimen combination (AZT/XTC/DTG or TDF/XTC/DTG or AZT/XTC/LPV/r) that participants were switched to after virological failure. Secondary exposures included participant baseline characteristics when starting second-line treatment, such as age, gender, active tuberculosis, most recent viral load, most recent CD4 count, and time on ART.

### Statistical analysis

We summarised participants' baseline demographic, clinical characteristics, and outcomes at 12 months follow-up. We conducted univariable and multivariable modified Poisson regression with robust standard errors adjusting for clustering by clinic^11^ to determine the risk ratios of retention in care and viral suppression at 12 months follow-up. In the regression analyses, we compared outcomes with the two newer DTG-based regimens (AZT/XTC/DTG and TDF/XTC/DTG) versus outcomes with the previous standard of care (AZT/XTC/LPVr). We also ran these models comparing the two DTG-based regimens against each other (AZT/XTC/DTG *vs* TDF/XTC/DTG). We calculated risk ratios and risk differences for the primary outcomes from these models. In the multivariable regression models, we adjusted for participant characteristics at baseline, namely age category, gender, active tuberculosis disease, and category for recent viral load. We excluded the most recent CD4 count, time on ART, and the baseline time period of switching to second-line treatment in the multivariable models, as including these variables resulted in out-of-bound predicted probabilities greater than one.^12^ Instead, we conducted sensitivity analyses of the effect of the ART regimen on each outcome, adjusted for only CD4 count, time on ART, and the baseline time-period of second-line switch, to demonstrate evidence of minimal confounding of the association between ART regimen and the primary outcomes by these variables. We conducted further sensitivity analyses excluding participants who changed their originally prescribed second-line regimen within 12 months after starting second-line treatment.

All exposure variables were complete except for the recent CD4 count at the time of switching to second-line treatment, which was missing for 163 participants. We did not impute missing CD4 counts as they might not be missing at random (as people who are more immunocompromised with low CD4 counts might be more likely to have CD4 counts taken). Instead, we created a missing category in the CD4 variable and included them in the analysis. We did all statistical analyses using R (version 4.2.0).

### Role of the funding source

The funder of the study had no role in study design, data collection, data analysis, data interpretation, or writing of the report.

## Results

From Dec 1, 2019, to Nov 30, 2020, 1672 people were recorded as switching to second-line ART after virological failure while receiving first-line ART at the study clinics (figure). We excluded 302 participants who were not previously receiving standard first-line regimens of TDF/XTC/EFV or TDF/XTC/NVP at the time of virological failure and 156 who were not switched to standard second-line regimens of AZT/XTC/LPV/r or AZT/XTC/DTG or TDF/XTC/DTG. Of the remaining 1214 participants included in this analysis, 689 (57%) were switched to AZT/XTC/LPV/r, 217 (18%) to AZT/XTC/DTG, and 308 (25%) to TDF/XTC/DTG second-line regimens.

**Figure:**
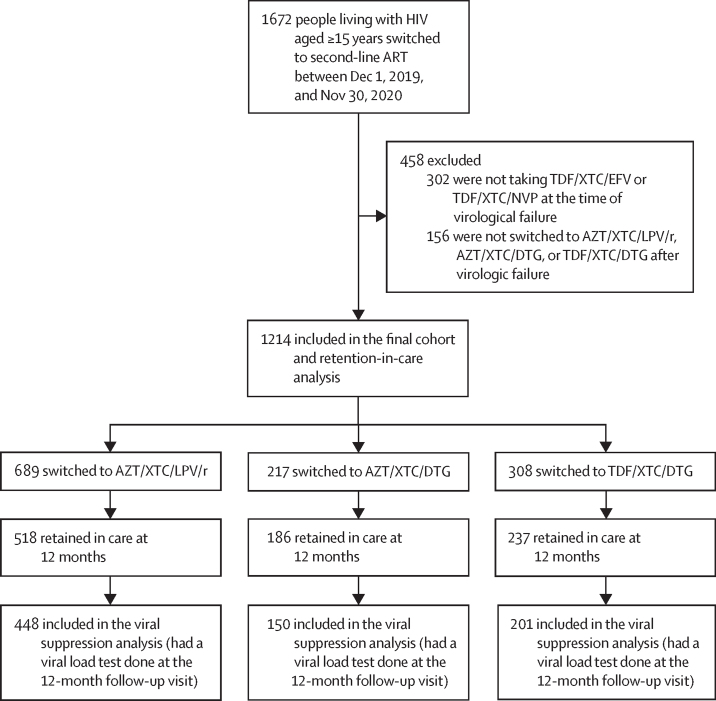
Flow diagram of participants receiving care at 59 clinics in South Africa ART=antiretroviral treatment. AZT=zidovudine. DTG=dolutegravir. EFV=efavirenz. LPV/r=ritonavir-boosted lopinavir. NVP=nevirapine. TDF=tenofovir disoproxil fumarate. XTC=emtricitabine or lamivudine.

Overall, the median age was 36 years (IQR 30–42), 729 (60%) were female, and 485 (40%) were male (table 1). Almost all participants previously received first-line TDF/XTC/EFV (n=1198, 99%). Age was similar between the three regimen groups, but there were more women in the AZT/XTC/LPV/r group (n=460, 67%) than in the AZT/XTC/DTG (n=108, 50%) and TDF/XTC/DTG (n=161, 52%) groups. The TDF/XTC/DTG group had more participants (n=155, 50%) with recent viral load at baseline lower than 10 000 copies per mL than the AZT/XTC/DTG (n=80, 37%) and AZT/XTC/LPV/r (n=260, 38%) groups. Time from the most recent viral load to second-line switch was a median of 50 days (IQR 28–95) in the AZT/XTC/LPV/r group, 49 days (28–102) in the AZT/XTC/DTG group, and 34 days (0–79) in the TDF/XTC/DTG group. A higher proportion of participants in the AZT/XTC/LPV/r (n=264, 38%) and AZT/XTC/DTG (n=94, 43%) groups had the most recent CD4 count of 200 cells per μL or less, compared with the TDF/XTC/DTG group (n=79, 26%).

**Table 1:** Baseline characteristics of people living with HIV who were switched to second-line ART after virological failure while receiving EFV-based* or NVP-based* first-line treatment

		**Overall (n=1214)**	**Second-line ART regimen combination**		
			AZT/XTC/LPV/r (n=689)	AZT/XTC/DTG (n=217)	TDF/XTC/DTG (n=308)
Age, years					
	Median age	36 (30–42)	35 (30–41)	37 (32–43)	36 (30–43)
	15–24 years	91 (7%)	54 (8%)	10 (5%)	27 (9%)
	25–34 years	429 (35%)	255 (37%)	71 (33%)	103 (33%)
	35–44 years	479 (39%)	274 (40%)	88 (41%)	117 (38%)
	≥45 years	215 (18%)	106 (15%)	48 (22%)	61 (20%)
Gender					
	Male	485 (40%)	229 (33%)	109 (50%)	147 (48%)
	Female	729 (60%)	460 (67%)	108 (50%)	161 (52%)
Known to be pregnant (females only)		14 (2%)	10 (2%)	1 (1%)	3 (2%)
Known to have tuberculosis		24 (2%)	14 (2%)	6 (3%)	4 (1%)
Time period of switch to second-line treatment					
	December, 2019–February, 2020	224 (18%)	190 (28%)	7 (3%)	27 (9%)
	March–May, 2020	324 (27%)	204 (30%)	54 (25%)	66 (21%)
	June–August, 2020	370 (30%)	165 (24%)	70 (32%)	135 (44%)
	September–November, 2020	296 (24%)	130 (19%)	86 (40%)	80 (26%)
Recent viral load before switch to second-line treatment					
	1000 to <10 000 copies per mL	495 (41%)	260 (38%)	80 (37%)	155 (50%)
	10 000 to <50 000 copies per mL	386 (32%)	220 (32%)	70 (32%)	96 (31%)
	50 000 to <100 000 copies per mL	133 (11%)	80 (12%)	32 (15%)	21 (7%)
	≥100 000 copies per mL	200 (16%)	129 (19%)	35 (16%)	36 (12%)
Median time since recent viral load before switch to second-line treatment, days		47 (26–92)	50 (28–95)	49 (28–102)	34 (0–79)
Median time since first high viral load before switch to second-line treatment, days		195 (140–276)	196 (139–282)	198 (141–300)	190 (140–252)
Recent CD4 count					
	Median count	238 (122–380)	226 (107–363)	205 (102–342)	289 (187–409)
	≤200 cells per μL	437 (36%)	264 (38%)	94 (43%)	79 (26%)
	201–350 cells per μL	307 (25%)	163 (24%)	51 (24%)	93 (30%)
	351–500 cells per μL	174 (14%)	90 (13%)	25 (12%)	59 (19%)
	>500 cells per μL	133 (11%)	72 (10%)	19 (9%)	42 (14%)
	Missing	163 (13%)	100 (15%)	28 (13%)	35 (11%)
Median time since recent CD4 count, days		400 (105–923)	402 (104–928)	273 (54–914)	434 (168–914)
Previous first-line ART before switch to second-line treatment					
	TDF/XTC/EFV	1198 (99%)	681 (99%)	215 (99%)	302 (98%)
	TDF/XTC/NVP	16 (1%)	8 (1%)	2 (1%)	6 (2%)
ART pick-up point					
	Main clinic	1192 (98%)	681 (99%)	215 (99%)	296 (96%)
	Central Chronic Medicines Dispensing and Distribution†	22 (2%)	8 (1%)	2 (1%)	12 (4%)
Time since ART initiation, years					
	Median time	2·9 (1·5–5·5)	2·9 (1·5–5·5)	3·5 (1·5–6·2)	2·6 (1·4–4·7)
	<2 year	446 (37%)	252 (37%)	72 (33%)	122 (40%)
	≥2 years	768 (63%)	437 (63%)	145 (67%)	186 (60%)

Data are n (%) or median (IQR). All percentages were calculated with the total number in the respective column headers as the denominators except where otherwise stated. ART=antiretroviral treatment. AZT=zidovudine. DTG=dolutegravir. EFV=efavirenz. LPV/r=ritonavir-boosted lopinavir. NVP=nevirapine. TDF=tenofovir disoproxil fumarate. XTC=emtricitabine or lamivudine.

* EFV-based or NVP-based first-line regimens were in combination with TDF plus XTC.

† Central Chronic Medicines Dispensing and Distribution included external or internal pickup points, spaced fast lanes, and adherence clubs.

During follow-up, 121 participants (10%) changed their originally prescribed second-line regimen after a median of 158 days (IQR 84–234; table 2). These regimen changes could be due to the ongoing transition to DTG-based second-line or to adverse reactions to originally prescribed regimens. By 12 months, 941 (78%) of 1214 were retained in care, 80 (7%) had transferred out to another clinic, 16 (1%) were known to have died, and 177 (15%) were lost to follow-up. The proportion of patients retained in care at 12 months was 75% (n=518) in participants receiving AZT/XTC/LPV/r, 86% (n=186) in those receiving AZT/XTC/DTG, and 77% (n=237) in those receiving TDF/XTC/DTG (table 3). After adjusting for potential confounders, retention in care at 12 months was more likely in participants receiving AZT/XTC/DTG than in those receiving AZT/XTC/LPV/r (adjusted risk ratio [aRR]=1·14, 95% CI 1·03–1·27, p=0·012; adjusted risk difference [aRD]=10·89%, 95% CI 2·01 to 19·78, p=0·016). Retention in care at 12 months did not differ between participants receiving TDF/XTC/DTG and those receiving AZT/XTC/LPV/r (aRR=1·01, 0·94–1·10, p=0·73; aRD=1·04%, –5·03 to 7·12, p=0·74). Retention in care was less likely in participants receiving TDF/XTC/DTG than AZT/XTC/DTG, although evidence for a difference was weak (aRR=0·89, 0·78–1·01, p=0·060; aRD=–9·85%, –20·33 to 0·63, p=0·066).

**Table 2:** Follow-up outcomes in people living with HIV who were switched to second-line ART after virological failure while receiving EFV-based* or NVP-based* first-line treatment

		**Overall (n=1214)**	**Second-line ART regimen combination**		
			AZT/XTC/LPV/r (n=689)	AZT/XTC/DTG (n=217)	TDF/XTC/DTG (n=308)
Second-line regimen-change within 12 months		121/1214 (10%)	59/689 (9%)	21/217 (10%)	41/308 (13%)
Median time to second-line regimen change within 12 months, days		158 (84–234)	146 (74–204)	182 (97–231)	160 (84–253)
Second-line regimen (of participants who changed regimen within 12 months)					
	AZT/XTC/LPV/r	17/121 (14%)	0	4/21 (19%)	13/41 (32%)
	AZT/XTC/DTG	35/121 (29%)	16/59 (27%)	0	19/41 (46%)
	TDF/XTC/DTG	26/121 (21%)	12/59 (20%)	14/21 (67%)	0
	Other	43/121 (36%)	31/59 (53%)	3/21 (14%)	9/41 (22%)
Follow-up outcome at 12 months					
	Lost to follow-up	177/1214 (15%)	112/689 (16%)	20/217 (9%)	45/308 (15%)
	Died	16/1214 (1%)	9/689 (1%)	4/217 (2%)	3/308 (1%)
	Transferred out to another clinic	80/1214 (7%)	50/689 (7%)	7/217 (3%)	23/308 (7%)
	Retained in care	941/1214 (78%)	518/689 (75%)	186/217 (86%)	237/308 (77%)
	Viral load test done at 12 months (of participants retained in care at 12 months)	799/941 (85%)	448/518 (86%)	150/186 (81%)	201/237 (85%)
	Median time to viral load test at 12 months (of participants retained in care at 12 months), days	357 (293–418)	362 (299–419)	342 (277–394)	357 (296–426)
Viral load at 12 months (of participants retained in care at 12 months with a viral load test done)					
	<50 copies per mL	420/799 (53%)	209/448 (47%)	89/150 (59%)	122/201 (61%)
	50–199 copies per mL	102/799 (13%)	61/448 (14%)	20/150 (13%)	21/201 (10%)
	200–999 copies per mL	75/799 (9%)	41/448 (9%)	20/150 (13%)	14/201 (7%)
	≥1000 copies per mL	202/799 (25%)	137/448 (31%)	21/150 (14%)	44/201 (22%)

Data are n/N (%) or median (IQR). All percentages were calculated with the total number in the respective column headers as the denominators except where otherwise stated. ART=antiretroviral treatment. AZT=zidovudine. DTG=dolutegravir. EFV=efavirenz. LPV/r=ritonavir-boosted lopinavir. NVP=nevirapine. TDF=tenofovir disoproxil fumarate. XTC=emtricitabine or lamivudine.

* EFV-based or NVP-based first-line regimens were in combination with TDF plus XTC.

**Table 3:** Univariable and multivariable Poisson regression models of factors associated with retention-in-care at 12 months in people living with HIV who were switched to second-line ART after virological failure while receiving EFV-based* or NVP-based* first-line treatment (n=1214)

	**Retention-in-care at 12 months**	**Unadjusted analysis**		**Adjusted analysis**†	
		RR (95% CI)	p value	RR (95% CI)	p value
**Second-line regimen**					
AZT/XTC/LPV/r	518/689 (75%)	1 (ref)	..	1 (ref)	..
AZT/XTC/DTG	186/217 (86%)	1·14 (1·03–1·27)	0·013	1·14 (1·03–1·27)	0·012
TDF/XTC/DTG	237/308 (77%)	1·02 (0·94–1·11)	0·63	1·01 (0·94–1·10)	0·73
**Age at baseline**					
15–24 years	67/91 (74%)	1 (ref)	..	1 (ref)	..
25–34 years	323/429 (75%)	1·03 (0·90–1·17)	0·71	1·03 (0·90–1·17)	0·71
35–44 years	377/479 (79%)	1·07 (0·94–1·22)	0·31	1·07 (0·95–1·22)	0·27
≥45 years	174/215 (81%)	1·10 (0·98–1·25)	0·11	1·10 (0·97–1·24)	0·14
**Gender**					
Male	373/485 (77%)	1 (ref)	..	1 (ref)	..
Female	568/729 (78%)	1·01 (0·95–1·08)	0·67	1·03 (0·98–1·10)	0·26
**Known tuberculosis status at baseline**					
No	925/1190 (78%)	1 (ref)	..	1 (ref)	..
Yes	16/24 (67%)	0·85 (0·63–1·15)	0·30	0·86 (0·64–1·15)	0·31
**Recent viral load at baseline**					
1000 to <10 000 copies per mL	399/495 (81%)	1 (ref)	..	1 (ref)	..
≥10 000 copies per mL	542/719 (75%)	0·93 (0·88–0·99)	0·031	0·94 (0·88–1·00)	0·042
**Recent CD4 count at baseline**					
≤200 cells per μL	338/437 (77%)	1 (ref)	..	..	..
201–350 cells per μL	235/307 (77%)	0·99 (0·91–1·08)	0·83	..	..
351–500 cells per μL	128/174 (74%)	0·95 (0·86–1·05)	0·33	..	..
>500 cells per μL	106/133 (80%)	1·03 (0·92–1·15)	0·59	..	..
Missing	134/163 (82%)	1·08 (0·99–1·17)	0·067	..	..
**Time on ART at baseline**					
<2 years	335/446 (75%)	1 (ref)	..	..	..
≥2 years	606/768 (79%)	1·05 (0·97–1·13)	0·21	..	..
**Time period of switch to second-line treatment**					
December, 2019–February, 2020	179/224 (80%)	..	..	..	..
March–May, 2020	255/324 (79%)	0·98 (0·91–1·05)	0·60	..	..
June–August, 2020	281/370 (76%)	0·95 (0·87–1·03)	0·21	..	..
September–November, 2020	226/296 (76%)	0·95 (0·87–1·04)	0·26		

Data are n/N (%), unless otherwise stated. ART=antiretroviral treatment. AZT=zidovudine. DTG=dolutegravir. EFV=efavirenz. LPV/r=ritonavir-boosted lopinavir. NVP=nevirapine. RR=risk ratio. TDF=tenofovir disoproxil fumarate. XTC=emtricitabine or lamivudine.

* EFV-based or NVP-based first-line regimens were in combination with TDF plus XTC.

† The primary exposure effect (retention-in-care at 12 months) is adjusted for all other variables in the table as potential confounders except recent CD4 count at baseline, years on ART at baseline, and time period of switch to second-line treatment.

Of 941 participants who were retained in care at 12 months, 799 (85%) had a viral load done at a median of 357 days (IQR 293–418; table 2). Of participants with a viral load test at 12 months, viral suppression (<50 copies per mL) was higher in those receiving AZT/XTC/DTG (n=89, 59%) and TDF/XTC/DTG (n=122, 61%) than AZT/XTC/LPV/r (n=209, 47%). Viral suppression at 12 months was more likely in participants receiving AZT/XTC/DTG (aRR=1·25, 1·06–1·47, p=0·0093; aRD=11·57%, 2·37 to 20·76, p=0·014) and in participants receiving TDF/XTC/DTG (aRR=1·30, 1·14–1·48, p<0·0001; aRD=14·16%, 7·14 to 21·18, p<0·0001) than in participants receiving AZT/XTC/LPV/r (table 4). Viral suppression at 12 months was similar between participants receiving TDF/XCT/DTG and those receiving AZT/XTC/DTG (aRR=1·04, 0·88–1·24, p=0·62; aRD=2·59%, –7·78 to 12·60, p=0·62). In a post-hoc sensitivity analysis presented as part of the supplementary results, viral suppression (defined as <1000 copies per mL) at 12 months was more likely in participants receiving AZT/XTC/DTG (86%, aRR=1·19, 1·07–1·32, p=0·0013; aRD=13·22%, 5·02 to 21·41, p=0·0016) and in participants receiving TDF/XTC/DTG (78%, aRR=1·11, 1·01–1·22, p=0·033; aRD=7·63%, 0·50 to 14·77, p=0·036) than in participants receiving AZT/XTC/LPV/r (69%; appendix 2 p 2). Viral suppression (<1000 copies per mL) at 12 months was similar between participants receiving TDF/XCT/DTG and those receiving AZT/XTC/DTG (aRR=0·93, 0·85–1·02, p=0·14; aRD=–5·58%, –13·12 to 1·95, p=0·15).

**Table 4:** Univariable and multivariable Poisson regression models of factors associated with viral suppression (<50 copies per mL) at 12 months in PLHIV who were switched to second-line ART after virological failure while receiving EFV* or NVP-based* first-line treatment (n=799)

	**Viral load at 12 months <50 copies per mL**	**Unadjusted analysis**		**Adjusted analysis**†	
		RR (95% CI)	p value	RR (95% CI)	p value
**Second-line regimen**					
AZT/XTC/LPV/r	209/448 (47%)	1 (ref)	..	1 (ref)	..
AZT/XTC/DTG	89/150 (59%)	1·22 (1·03–1·46)	0·022	1·25 (1·06–1·47)	0·0093
TDF/XTC/DTG	122/201 (61%)	1·31 (1·15–1·49)	<0·0001	1·30 (1·14–1·48)	<0·0001
**Age at baseline**					
15–24 years	21/56 (38%)	1 (ref)	..	1 (ref)	..
25–34 years	153/282 (54%)	1·46 (0·99–2·14)	0·056	1·50 (1·01–2·21)	0·043
35–44 years	159/308 (52%)	1·37 (0·91–2·06)	0·13	1·45 (0·96–2·17)	0·075
≥45 years	87/153 (57%)	1·55 (1·06–2·27)	0·024	1·58 (1·07–2·33)	0·022
**Gender**					
Male	156/313 (50%)	1 (ref)	..	1 (ref)	..
Female	264/486 (54%)	1·10 (0·98–1·23)	0·11	1·12 (1·00–1·25)	0·053
**Known tuberculosis status at baseline**					
No	415/784 (53%)	1 (ref)	..	1 (ref)	..
Yes	5/15 (33%)	0·66 (0·30–1·45)	0·30	0·69 (0·32–1·48)	0·340
**Recent viral load at baseline**					
1000 to <10 000 copies per mL	198/337 (59%)	1 (ref)	..	1 (ref)	..
≥10 000 copies per mL	222/462 (48%)	0·83 (0·73–0·93)	0·0018	0·85 (0·76–0·96)	0·0075
**Recent CD4 count at baseline**					
≤200 cells per μL	153/292 (52%)	1 (ref)	..	..	..
201–350 cells per μL	96/194 (49%)	0·95 (0·81–1·11)	0·49	..	..
351–500 cells per μL	66/108 (61%)	1·14 (0·94–1·39)	0·18	..	..
>500 cells per μL	42/90 (47%)	0·90 (0·66–1·23)	0·51	..	..
Missing	63/115 (55%)	1·04 (0·83–1·31)	0·73	..	..
**Years on ART at baseline**					
<2 year	156/286 (55%)	1 (ref)	..	..	..
≥2 years	264/513 (51%)	0·95 (0·82–1·10)	0·48	..	..
**Time period of switching to second-line treatment**					
December, 2019–February, 2020	81/159 (51%)	..	..	..	..
March–May, 2020	113/217 (52%)	1·04 (0·87–1·24)	0·68	..	..
June–August, 2020	125/244 (51%)	1·02 (0·86–1·21)	0·85	..	..
September–November, 2020	101/179 (56%)	1·09 (0·91–1·31)	0·34	..	..

Data are n/N (%), unless otherwise stated. ART=antiretroviral treatment. AZT=zidovudine. DTG=dolutegravir. EFV=efavirenz. LPV/r=ritonavir-boosted lopinavir. NVP=nevirapine. PLHIV=people living with HIV. RR=risk ratio. TDF=tenofovir disoproxil fumarate. XTC=emtricitabine or lamivudine.

* Efavirenz or nevirapine based first-line regimens were in combination with TDF plus XTC.

† The primary exposure effect (viral suppression at 12 months) is adjusted for all other variables in the table as potential confounders except recent CD4 count at baseline, years on ART at baseline, and time period of switch to second-line treatment.

Appendix 2 (pp 3–5) shows minimal confounding of retention in care and viral suppression outcomes by recent baseline CD4 count, time on ART, and baseline period of second-line switch. Results show that the outcomes of retention in care and viral suppression were consistent with the primary analysis after excluding participants who changed their originally prescribed second-line regimens within 12 months after starting second-line treatment (appendix 2 pp 6–8).

## Discussion

In this retrospective cohort study with routine data from 59 ART clinics in South Africa, we found that second-line DTG-based regimens (AZT/XTC/DTG and TDF/XTC/DTG) were associated with similar or better retention in care and better viral suppression than the previously recommended second-line AZT/XTC/LPV/r regimen.

We evaluated retention in care at 12 months because drug tolerability is known to affect adherence^13^ and retention in care.^14^ We saw higher retention in care with AZT/XTC/DTG than with AZT/XTC/LPV/r, consistent with the favourable safety profile of DTG-based compared with protease-inhibitor-based regimens for second-line treatment shown in the DAWNING^5^ and NADIA^15^ trials and generally reported during first-line ART.^16^, ^17^, ^18^ Observed retention in care was lower with TDF/XTC/DTG (77%) than with AZT/XTC/DTG (86%), although in the multivariable analysis the evidence for a difference between the two regimens was weak (aRD=–9·85%, p=0·066). We expected similar rates between the two regimens as TDF is slightly more tolerable than AZT.^19^, ^20^ This observed difference could be a result of unmeasured confounding. For example, people who received TDF/XTC/DTG after virological failure might have been put on this regimen in error as part of the transition to first-line DTG or were more likely to have anaemia (a contraindication to AZT^4^), which is not recorded in TIER.net. They might, therefore, differ from those receiving AZT/XTC/DTG (eg, they might not have received enhanced adherence counselling or those with anaemia might be less clinically well), which could explain the lower retention in care seen in this group. Hence, we caution against interpreting our results to indicate superior retention with AZT than TDF during second-line ART. The NADIA trial showed low rates of adverse events leading to second-line treatment discontinuation in the TDF-based (n=2, 1·0%) and the AZT-based (n=3, 1·0%) groups.^15^

The DAWNING trial^5^ is the only clinical trial directly comparing the efficacy of DTG versus LPV/r for second-line ART. The trial enrolled 624 people living with HIV aged 18 years or older with virological failure during first-line treatment, who were randomly assigned to receive DTG (n=312) or LPV/r (n=312) second-line regimens plus two NRTIs, with at least one being fully active based on results from HIV drug-resistance testing. More participants reported high scores for medication adherence in the DTG-based group than in the LPV/r group (67% *vs* 56%), and fewer participants reported treatment-related adverse events in the DTG-based group than in the LPV/r group (16% *vs* 38%).^5^ There were also fewer adverse events leading to treatment discontinuation in the DTG group (3%) than in the LPV/r group (6%), which might explain the improved retention in care that we noted with AZT/XTC/DTG versus AZT/XTC/LPV/r.^5^ In the primary intention-to-treat analysis of the DAWNING trial, viral suppression (viral load <50 copies per mL) at 48 weeks was higher in the DTG group (84%) than in the LPV/r group (70%; aRD=13·8%; 95% CI 7·3–20·3), which is consistent with our findings.^5^

We found four clinical trials assessing the efficacy of recycling TDF in a second-line regimen. The NADIA trial used a 2 × 2 factorial design to randomise people living with HIV who had virological failure during first-line treatment to receive either second-line DTG or second-line lopinavir-boosted darunavir with either TDF or AZT.^6^ Recycling TDF for second-line treatment was non-inferior to switching to zidovudine for viral suppression (viral load <400 copies per mL) at 48 weeks.^6^ Consistent with results from the NADIA trial, we found no difference between TDF/XTC/DTG and AZT/XTC/DTG for viral suppression at less than 50 copies per mL at 48 weeks.^6^ The smaller single-arm ARTIST trial done in 62 participants showed 74% of participants had viral suppression (<50 copies per mL) at 48 weeks with TDF/XTC/DTG during second-line treatment.^21^ Preliminary results from the VISEND^22^ and D2EFT^23^ trials also showed TDF/XTC/DTG as being non-inferior to LPV/r or atazanavir in the VISEND trial and darunavir in the D2EFT trial. In this routine-care setting of our study, TDF/XTC/DTG was associated with better viral suppression than AZT/XTC/LPV/r.

Viral suppression rates in these trials are generally higher than the rates we found in routine care, probably because of better treatment adherence and monitoring among participants in clinical trials.^24^ But differences in cohort baseline virological failure and post-baseline viral suppression thresholds might also be responsible for the different outcomes. Although the DAWNING^5^ trial used a viral suppression of less than 50 copies per mL, it included participants with a baseline viral load between 400 copies per mL and less than 1000 copies per mL, which is different to the guideline-defined threshold of 1000 copies per mL or higher used in our cohort. The NADIA^6^ trial used a baseline viral load of 1000 copies per mL or higher, as we did, but viral suppression was defined at less than 400 copies per mL. The VISEND^22^ trial included participants with a baseline viral load of 1000 copies per mL or higher and used a viral suppression threshold of less than 1000 copies per mL. The resulting viral suppression of less than 1000 copies per mL at 12 months (82% with TDF/XTC/DTG and 76% with AZT/3TC plus LPV/r or atazanavir/r)^22^ in the VISEND trial was similar to what we noted in our post-hoc sensitivity analysis with the same thresholds (78% with TDF/XTC/DTG, 69% with AZT/XTC/LPV/r, and 86% with AZT/XTC/DTG).

Although clinical trials have assessed the use of second-line DTG regimens after virological failure with first-line NNRTI-based regimens, to the best of our knowledge, this is the first cohort study among people living with HIV in a routine-care setting, where factors such as outcomes, clinical management, and participants probably differ from clinical trials. We used guideline-defined virological failure, viral suppression, and retention in care and adjusted for the effects of baseline characteristics when switching to second-line treatment. Our findings support WHO's recommendation of DTG for second-line ART in adults with treatment failure on first-line NNRTI-based regimens. Although the rollout of first-line DTG progresses well, a substantial proportion of people remain on non-DTG-based second-line ART.^25^ Furthermore, while the 2023 WHO HIV policy adoption and implementation update confirms that DTG is currently available in second-line regimens in 89 (77%) of 116 LMICs, only 47 (41%) countries have it as the main or preferred option,^26^ which might explain why a significant number of people remain on non-DTG-based second-line regimens. Our findings are relevant as they provide further impetus for promoting changes to national guidelines in these settings to accelerate the transition from LPV/r to DTG-based second-line regimens, which is likely to require several years. Our findings highlight the performance of DTG for second-line ART in routine care and implementation bottlenecks. Overall, 12-month outcomes with all second-line regimens were poorer in this study than in clinical trials. Of the 1214 people in the cohort, about a third (n=420, 35%) achieved programmatic retention in care and viral suppression (<50 copies per mL) at 12 months, with 379 (47%) of 799 people retained in care still having viraemia on second-line treatment. High levels of ongoing viraemia on these second-line regimens could lead to the emergence of mutations responsible for DTG resistance.^15^, ^27^ Outcomes were also poorer in younger people and those with higher baseline viral load, indicating that early viraemia during second-line ART is probably due to ongoing poor adherence rather than resistance. This finding highlights the need to improve interventions such as adherence counselling in the HIV care cascade during second-line treatment, to better support medication adherence, as regimen choice is only one factor for improving HIV treatment outcomes. Dedicated adherence clinics or community ART delivery programmes^28^ and other evidence-based adherence support strategies, particularly among younger people and those at higher risk of poor outcomes, could improve outcomes during second-line treatment.

Furthermore, WHO recommends the substitution of TDF, a common drug in most first-line regimens in LMICs, with AZT when switching to second-line treatment to ensure having an active NRTI backbone because of limited resistance testing for selecting appropriate NRTIs.^1^, ^4^ However, based on results from the NADIA trial suggesting non-inferiority of recycling TDF instead of switching to AZT and the availability of TLD as a fixed dose combination, TDF/XTC/DTG is considered an easily implementable regimen in most LMICs.^7^ Our findings have provided further assurance regarding these assertions with evidence from routine care. This finding is, therefore, also relevant to other resource-limited settings where resistance testing is not routinely done to guide the selection of NRTIs for second-line treatment.

The absence of resistance testing in our cohort means we could not classify virological failures on the basis of drug-resistance mutations or ongoing poor adherence. Likewise, we could not determine the effect of pre-switch resistance on outcomes, but a substudy of the EARNEST trial^29^ showed that pre-switch NRTI resistance was rather associated with viral suppression after switching to protease-inhibitor-based second-line ART. In subanalyses of the DAWNING^27^ and NADIA^15^ trials, there were a few cases of emergent resistance among people receiving DTG second-line, but none in those receiving protease-inhibitor-based regimes such as LPV/r second-line, which means that among the significant proportion of people who did not suppress in our study, the likelihood of DTG-resistance might be higher in the DTG groups than in the protease-inhibitor group.

Our analysis had some potential limitations. First, we used data from only one district in South Africa, which might limit the generalisability of the findings. Our sample size was similar to clinical trials but might not be large enough to detect smaller effect sizes. Second, we only assessed 12-month outcomes, and evaluating longer-term follow-up will be important in future analyses. Third, in a new era of DTG, clinicians and nurses might have selected specific people living with HIV for DTG treatment who were more likely to have better outcomes, which might explain why a higher proportion of participants receiving TDF/XTC/DTG in our study had a baseline viral load lower than 10 000 copies per mL. We therefore adjusted for baseline viral load and other relevant baseline characteristics, but we cannot rule out potential unmeasured confounders. Fourth, we were unable to include the recent CD4 count, time on ART, and the baseline period of switch to second-line treatment in the multivariable analyses as it led to overfitted models with predicted probabilities exceeding one. Therefore, we evaluated the impact of baseline CD4 count, years on ART, and the baseline period of switch to second-line treatment in supplementary analyses, which showed minimal evidence of confounding of the association between DTG use and the primary outcomes. Finally, the baseline period of our study partly coincided with the COVID-19 pandemic, which might have disrupted access to HIV care. However, our results showed that the observed primary outcomes were similar across the baseline period categories with minimal evidence of confounding.

Despite these limitations, our findings provide reassurance that in routine-care settings, DTG can be used successfully in second-line regimens in a resource-limited setting such as South Africa. Further impetus is required to ensure the availability of DTG in second-line regimens in LMICs, both for people who are failing first-line NNRTI-based regimens and for people on second-line LPVr-based regimens who could also benefit from being transitioned to DTG.

## Equitable partnership declaration

## Data sharing

We cannot publicly share the data used for this analysis because of the legal and ethical requirements regarding the use of routinely collected clinical data in South Africa. Interested parties can request access to the data from the eThekwini Municipality Health Unit and the South African National Department of Health TB/HIV Information System (contact details obtainable upon request to JD).

## Declaration of interests

RJL is a recipient of research awards from the National Institute of Allergy and Infectious Diseases of the National Institutes of Health under award numbers R01AI152772 and R01AI167699. These awards are for projects relating to the monitoring of HIV drug resistance (focused on dolutegravir resistance) and evaluation of management strategies for people with virological failure on dolutegravir-containing regimens. All other authors declare no competing interests.

## Supplementary Materials

isiZulu translation of the abstract

Supplementary appendix 2

Equitable Partnership Declaration
