# Supplementary material for: Clinical outcomes with second-line dolutegravir in people with virological failure on first-line non-nucleoside reverse transcriptase inhibitor-based regimens in South Africa: a retrospective cohort study
Source: Lancet Glob Health. 2023 Dec 21;12(2):e282–91. doi: 10.1016/S2214-109X(23)00516-8 (PMC10805003; doi:10.1016/S2214-109X(23)00516-8)
Supplement: isiZulu translation of the abstract [file mmc1.pdf]

THE LANCET
Global Health

Supplementary appendix 1

This translation in isiZulu was submitted by the authors and we reproduce it as supplied. It has not been peer reviewed. *The Lancet's* editorial processes have only been applied to the original in English, which should serve as reference for this manuscript.

Lokhu kuhunyushwa ngolimi lwesiZulu kwethulwe ngababhali futhi siyikhiqiza kabusha njengoba kuhlinzekiwe. Akuzange kubuyekezwe ontanga. Izinqubo zokuhlela *zeLancet* zisetshenziswe kuphela koqobo lwesiNgisi, okufanele lusebenze njengesethenjwa salo mbhalo.

Supplement to: Asare K, Sookrajh Y, van der Molen J, et al. Clinical outcomes with second-line dolutegravir in people with virological failure on first-line non-nucleoside reverse transcriptase inhibitor-based regimens in South Africa: a retrospective cohort study. *Lancet Glob Health* 2023; published online Dec 21. https://doi.org/10.1016/S2214-109X(23)00516-8.

Imiphumela yezokwelashwa ngomugqa wesibili we-dolutegravir kubantu okuhluleke ukucindezeleka kwegciwane kumugqa wokuqala we-non-nucleoside reverse transcriptase inhibitor (NNRTI) eNingizimu Afrika: ucwaningo lweqembu olubuyela emuva.

ISIFINYEZO

Isendulelo. I-Dolutegravir (DTG) inconyelwe ukwelashwa ngezidambisi gciwane (ART) zomugqa wesibili emuva kokwehluleka kwe-ART ezigabeni ezisuselwe kumugqa wokuqala we-non-nucleoside reverse transcriptase inhibitor (NNRTI) ukucindezela umthamo wegciwane egazini kubantu abaphila negciwane lesandulela ngculaza emazweni anemali engenayo ephansi naphakathi. Siqhathanise ukusebenza kahle kwe-DTG uma iqhathaniswa nohlobo lwe-lopinavir olukhuthazwe nge-ritonavir olwalunconywe ngaphambilini lokwelashwa komugqa wesibili eNingizimu Afrika.

Izindlela. Kulolu cwaningo lweqembu lokubuka emuva, sisebenzise idatha engahlonzwanga, eqoqwe njalo ezikhungweni zezempilo eziyisisekelo ezingu-59 kuMasipala waseThekwini, KwaZulu-Natal, eNingizimu Afrika. Sifake abantu abaphila negciwane lesandulela ngculaza abaneminyaka engu-15 noma ngaphezulu abano mthamo wegciwane egazini owahluleka ukucindezeleka (okuchazwa njengenani legciwane egazini elilandelanayo lika ≥1000 amakhophi nge-mL okungenani izinsuku ezingu-56 ngokuhlukana) ku-ART yomugqa wokuqala esekelwe ku-NNRTI equkethe i-tenofovir disoproxil fumarate (TDF) futhi bashintshelwa ku-ART yomugqa wesibili. Imiphumela yethu eyinhloko kwaba ukugcinwa ekunakekelweni nokucindezelwa kwegciwane (<50 amakhophi nge-mL) ezinyangeni eziyi-12 ngemva kokuqala ukwelashwa nge-ART yomugqa wesibili. Sisebenzise amamodeli we-Poisson regression ukuze siqhathanise lemiphumela phakathi kwezigaba zomugqa wesibili (<50 amakhophi/ml) ngemva kokuqala kwezigaba zomugqa wesibili we-(zidovudine (AZT)/ emtricitabine noma lamivudine (XTC)/ DTG; TDF /XTC/DTG kanye ne-AZT/XTC/LPV/r.

Imiphumela. Sifake ababambiqhaza abangu-1214 ocwaningweni lwethu, abangu-729 (60%) kubo bekungabesifazane, abangu-485 (40%) kungabesilisa, futhi iminyaka yabo yobudala ephakathi yayiyiminyaka engu-36 (IQR 30-42). Abangu-689 (57%) bashintshelwe ku-AZT/XTC/LPV/r, abangu-217 (18%) ku-AZT/XTC/DTG kanye no-308 (25%) oyiswe ku-TDF/XTC/DTG. Uma kuqhathaniswa ne-AZT/XTC/LPV/r (75%),ukugcinwa ekunakekelweni bekuphakeme nge-AZT/XTC/DTG (86%, adjusted risk ratio [aRR]=1·14,

95% CI 1·03 -1·27; adjusted risk difference [aRD]= 10·89%, 95% CI 2·01 kuya ku 19·78) kodwa kufanange-TDF/XTC/DTG (77%, aRR=1·01, 0·94-1·10; aRD= 1·04%, -5·03 kuya ku7·12). Ukugcinwa ekunakekelweni okubhekiwe bekuphansi nge-TDF/XTC/DTG kune-AZT/XTC/DTG, nakuba ubufakazi bomehluko kwi- multivariable analysis babubuthakathaka (aRR=0·89, 0·78-1 · 01, p=0·060; aRD=-9·85%, -20·33 kuya ku-0·63, p=0·066). Kuba bambiqhaza abangu-799 abagcinwe ekunakekelweni ngokuhlolwa kwenani legciwane egazini lezinyanga eziyi-12, ukucindezelwa kwegciwane kwakuphezulu nge-AZT/XTC/DTG (59%;aRR=1 ·25, 1 ·06-1· 47; aRD=11·57%, 2·37 kuya ku 20·76) nangaphezulu nge-TDF/XTC/DTG (61%;aRR=1·30, 1·14-1· 48; aRD=14·16%, 7·14 kuya ku 21·18) kune-AZT/XTC/LPV/r (47%).

Incazelo. Lemiphumela etholakele ekunakekelweni okujwayelekile kusekela ukuqaliswa okuqhubekayo kwesincomo se-WHO sokusebenzisa i-DTG esikhundleni se-LPV/r kubantu abaphila negciwane lesandulela ngculaza abahlangabezana nokwehluleka ukucindezeleka komthamo wegciwane egazini ngesikhathi bethola i-ART yomugqa wokuqala esekelwe ku-NNRTI.
