# Supplementary material for: Clinical outcomes with second-line dolutegravir in people with virological failure on first-line non-nucleoside reverse transcriptase inhibitor-based regimens in South Africa: a retrospective cohort study
Source: Lancet Glob Health. 2023 Dec 21;12(2):e282–91. doi: 10.1016/S2214-109X(23)00516-8 (PMC10805003; doi:10.1016/S2214-109X(23)00516-8)
Supplement: Supplementary appendix 2 [file mmc2.pdf]

THE LANCET
Global Health

Supplementary appendix 2

This appendix formed part of the original submission and has been peer reviewed. We post it as supplied by the authors.

Supplement to: Asare K, Sookrajh Y, van der Molen J, et al. Clinical outcomes with second-line dolutegravir in people with virological failure on first-line non-nucleoside reverse transcriptase inhibitor-based regimens in South Africa: a retrospective cohort study. *Lancet Glob Health* 2023; published online Dec 21. https://doi.org/10.1016/S2214-109X(23)00516-8.

Supplementary Appendix for *Clinical outcomes after the introduction of dolutegravir for second-line antiretroviral therapy in South Africa*

Appendix A: Supplementary Tables

Table S 1. Post-hoc sensitivity analysis: Univariable and multivariable Poisson regression models of factors associated with viral suppression (< 1000 copies/ml) at 12 months (N = 799)

Table S 2. Potential confounding effects of CD4 count and Years on ART on the risk ratios of 12-month retention-in-care (N = 1214)

Table S 3. Potential confounding effects of CD4 count and Years on ART on the risk ratios of 12-month viral suppression (< 50 copies/ml) (N = 799)

Table S 4. Potential confounding effects of CD4 count and Years on ART on the risk ratios of 12-month viral suppression (< 1000 copies/ml) (N = 799)

Table S 5. Retention-in-care at 12 months excluding 121 participants who changed their originally prescribed second-line regimen within 12 months after baseline (N = 1093)

Table S 6. Viral suppression (<50 copies/ml) at 12 months excluding 121 participants who changed their originally prescribed second-line regimen within 12 months after baseline (N = 713)

Table S 7. Viral suppression (<1000 copies/ml) at 12 months excluding 121 participants who changed their originally prescribed second-line regimen within 12 months after baseline (N = 713)

**Table S 1. Post-hoc sensitivity analysis: Univariable and multivariable Poisson regression models of factors associated with viral suppression (< 1000 copies/ml) at 12 months (N = 799)**						
Variable	Level	Viral load at 12 months < 1000 copies/ml n/N (%)	Unadjusted RR (95% CI)	P value	Adjusted RR[b] (95% CI)	P value
Second-line regimen	AZT/XTC/LPV/r	311/448 (69·4)	1	-	1	-
	AZT/XTC/DTG	129/150 (86·0)	1·18 (1·07-1·31)	0·0013	1·19 (1·07-1·32)	0·0013
	TDF/XTC/DTG	157/201 (78·1)	1·13 (1·02-1·24)	0·015	1·11 (1·01-1·22)	0·033
Age at baseline	15-24	31/56 (55·4)	1	-	1	-
	25-34	207/282 (73·4)	1·32 (1·02-1·70)	0·035	1·35 (1·04-1·74)	0·024
	35-44	240/308 (77·9)	1·38 (1·04-1·82)	0·024	1·40 (1·06-1·84)	0·019
	45+	119/153 (77·8)	1·42 (1·09-1·85)	0·0096	1·37 (1·05-1·81)	0·022
Gender	Male	243/313 (77·6)	1	-	1	-
	Female	354/486 (72·8)	0·95 (0·88-1·02)	0·14	0·95 (0·89-1·03)	0·22
Known tuberculosis status at baseline	No	588/784 (75·0)	1	-	1	-
	Yes	9/15 (60·0)	0·82 (0·54-1·25)	0·36	0·81 (0·55-1·22)	0·32
Recent viral load (copies/ml) at baseline	1000 to < 10000	276/337 (81·9)	1	-	1	-
	≥ 10000	321/462 (69·5)	0·86 (0·79-0·93)	<0·0001	0·85 (0·78-0·92)	<0·0001
Recent CD4 count (cells/µL) at baseline	≤ 200	220/292 (75·3)	1	-	1	-
	201–350	142/194 (73·2)	0·98 (0·90-1·06)	0·58	-	-
	351–500	88/108 (81·5)	1·07 (0·94-1·22)	0·30	-	-
	> 500	61/90 (67·8)	0·91 (0·76-1·09)	0·31	-	-
	Missing	86/115 (74·8)	1·00 (0·88-1·14)	0·99	-	-
Years on ART at baseline	< 2 year	220/286 (76·9)	1	-	-	-
	≥ 2 years	377/513 (73·5)	0·96 (0·89-1·03)	0·27	-	-
Time-period of switching to second-line treatment	Dec19-Feb20	123/159 (77·4)	-	-	-	-
	Mar20-May20	163/217 (75·1)	0·98 (0·88-1·08)	0·65	-	-
	Jun20-Aug20	170/244 (69·7)	0·91 (0·81-1·03)	0·13	-	-
	Sep20-Nov20	141/179 (78·8)	1·00 (0·90-1·11)	0·98		
Data are n/N (%), unless otherwise stated. [a]Efavirenz or nevirapine based first-line regimens were in combination with TDF plus XTC. [b]The primary exposure effect (viral suppression at 12 months) is adjusted for all other variables in the table as potential confounders. ART = Antiretroviral treatment, AZT = Zidovudine, DTG = Dolutegravir, EFV = Efavirenz, LVP/r = Lopinavir-ritonavir, µl = Microliter, ml = Milliliter, NVP = Nevirapine, PLHIV = People living with HIV, RR = Risk ratio, TDF = Tenofovir disoproxil fumarate, XTC = Emtricitabine or Lamivudine.						

Table S 2. Potential confounding effects of CD4 count and Years on ART on the risk ratios of 12-month retention-in-care (N = 1214)						
Variable	Level	Retention-in-care at 12 months n/N (%)	Unadjusted RR (95% CI)	P value	Adjusted RR[a] (95% CI)	P value
Second-line regimen	AZT/XTC/LPV/r	518/689 (75·2)	1	-	1	-
	AZT/XTC/DTG	186/217 (85·7)	1·14 (1·03-1·27)	0·013	1·14 (1·02-1·27)	0·016
	TDF/XTC/DTG	237/308 (76·9)	1·02 (0·94-1·11)	0·63	1·03 (0·95-1·12)	0·52
Recent CD4 count (cells/μL) at baseline	≤ 200	338/437 (77·3)	1	-	1	-
	201–350	235/307 (76·5)	0·99 (0·91-1·08)	0·83	0·99 (0·90-1·08)	0·76
	351–500	128/174 (73·6)	0·95 (0·86-1·05)	0·33	0·95 (0·86-1·04)	0·28
	> 500	106/133 (79·7)	1·03 (0·92-1·15)	0·59	1·03 (0·92-1·15)	0·63
	Missing	134/163 (82·2)	1·08 (0·99-1·17)	0·067	1·08 (1·00-1·17)	0·049
Years on ART at baseline	< 2 year	335/446 (75·1)	1	-	1	-
	≥ 2 years	606/768 (78·9)	1·05 (0·97-1·13)	0·21	1·05 (0·97-1·14)	0·19
Time-period of switching to second-line treatment	Dec19-Feb20	179/224 (79·9)	1	-	1	-
	Mar20-May20	255/324 (78·7)	0·98 (0·91-1·05)	0·59	0·95 (0·89-1·02)	0·17
	Jun20-Aug20	281/370 (75·9)	0·95 (0·87-1·03)	0·21	0·90 (0·83-0·99)	0·026
	Sep20-Nov20	226/296 (76·4)	0·95 (0·87-1·04)	0·26	0·90 (0·83-0·99)	0·022

Data are n/N (%), unless otherwise stated. [a]The primary exposure effect (retention-in-care at 12 months) is adjusted for CD4 count and Years on ART at baseline. ART = Antiretroviral treatment, AZT = Zidovudine, DTG = Dolutegravir, LVP/r = Lopinavir-ritonavir, TDF = Tenofovir disoproxil fumarate, RR = Risk ratio, XTC = Emtricitabine or Lamivudine.

Table S 3. Potential confounding effects of CD4 count and Years on ART on the risk ratios of 12-month viral suppression (< 50 copies/ml) (N = 799)

Variable	Level	Viral load at 12 months < 50 copies/ml n/N (%)	Unadjusted RR (95% CI)	P value	Adjusted RR[a] (95% CI)	P value
Second-line regimen	AZT/XTC/LPV/r	209/448 (46·7)	1	-	1	-
	AZT/XTC/DTG	89/150 (59·3)	1·22 (1·03-1·46)	0·022	1·23 (1·04-1·47)	0·017
	TDF/XTC/DTG	122/201 (60·7)	1·31 (1·15-1·49)	<0·0001	1·31 (1·15-1·49)	<0·0001
Recent CD4 count (cells/μL) at baseline	≤ 200	153/292 (52·4)	1	-	1	-
	201–350	96/194 (49·5)	0·95 (0·81-1·11)	0·49	0·94 (0·80-1·10)	0·43
	351–500	66/108 (61·1)	1·14 (0·94-1·39)	0·18	1·13 (0·92-1·38)	0·23
	> 500	42/90 (46·7)	0·90 (0·66-1·23)	0·51	0·88 (0·64-1·20)	0·42
	Missing	63/115 (54·8)	1·04 (0·83-1·31)	0·73	1·05 (0·84-1·31)	0·69
Years on ART at baseline	< 2 year	156/286 (54·5)	1	-	1	-
	≥ 2 years	264/513 (51·5)	0·95 (0·82-1·10)	0·48	0·94 (0·81-1·10)	0·47
Time-period of switching to second-line treatment	Dec19-Feb20	81/159 (50·9)	1	-	1	-
	Mar20-May20	113/217 (52·1)	1·04 (0·87-1·24)	0·68	0·98 (0·82-1·18)	0·83
	Jun20-Aug20	125/244 (51·2)	1·02 (0·86-1·21)	0·85	0·92 (0·77-1·09)	0·33
	Sep20-Nov20	101/179 (56·4)	1·09 (0·91-1·31)	0·34	0·98 (0·79-1·20)	0·89

Data are n/N (%), unless otherwise stated. [a]The primary exposure effect (viral suppression at 12 months) is adjusted for CD4 count and Years on ART at baseline. ART = Antiretroviral treatment, AZT = Zidovudine, DTG = Dolutegravir, LVP/r = Lopinavir-ritonavir, TDF = Tenofovir disoproxil fumarate, RR = Risk ratio, XTC = Emtricitabine or Lamivudine.

Table S 4. Potential confounding effects of CD4 count and Years on ART on the risk ratios of 12-month viral suppression (< 1000 copies/ml) (N = 799)						
Variable	Level	Viral load at 12 months < 1000 copies/ml n/N (%)	Unadjusted RR (95% CI)	P value	Adjusted RR[a] (95% CI)	P value
Second-line regimen	AZT/XTC/LPV/r	311/448 (69·4)	1	-	1	-
	AZT/XTC/DTG	129/150 (86·0)	1·18 (1·07-1·31)	0·0013	1·19 (1·07-1·32)	0·0013
	TDF/XTC/DTG	157/201 (78·1)	1·13 (1·02-1·24)	0·015	1·13 (1·02-1·25)	0·018
Recent CD4 count (cells/µL) at baseline	≤ 200	220/292 (75·3)	1	-	1	-
	201–350	142/194 (73·2)	0·98 (0·90-1·06)	0·58	0·98 (0·90-1·07)	0·67
	351–500	88/108 (81·5)	1·07 (0·94-1·22)	0·30	1·08 (0·94-1·23)	0·27
	> 500	61/90 (67·8)	0·91 (0·76-1·09)	0·31	0·91 (0·76-1·09)	0·30
	Missing	86/115 (74·8)	1·00 (0·88-1·14)	0·99	1·01 (0·88-1·15)	0·93
Years on ART at baseline	< 2 year	220/286 (76·9)	1	-	1	-
	≥ 2 years	377/513 (73·5)	0·96 (0·89-1·03)	0·26	0·95 (0·88-1·03)	0·22
Time-period of switching to second-line treatment	Dec19-Feb20	123/159 (77·4)	1	-	1	-
	Mar20-May20	163/217 (75·1)	0·98 (0·88-1·08)	0·65	0·94 (0·85-1·04)	0·230
	Jun20-Aug20	170/244 (69·7)	0·91 (0·81-1·03)	0·13	0·85 (0·75-0·96)	0·008
	Sep20-Nov20	141/179 (78·8)	1·00 (0·90-1·11)	0·98	0·92 (0·82-1·02)	0·13

Data are n/N (%), unless otherwise stated. [a]The primary exposure effect (viral suppression at 12 months) is adjusted for CD4 count and Years on ART at baseline. ART = Antiretroviral treatment, AZT = Zidovudine, DTG = Dolutegravir, LVP/r = Lopinavir-ritonavir, TDF = Tenofovir disoproxil fumarate, RR = Risk ratio, XTC = Emtricitabine or Lamivudine.

Table S 5. Univariable and multivariable Poisson regression models of factors associated with retention-in-care at 12 months excluding 121 participants who changed their originally prescribed second-line regimen within 12 months after baseline (N = 1093)						
Variable	Level	Retention-in-care at 12 months n/N (%)	Unadjusted RR (95% CI)	P value	Adjusted RR[a] (95% CI)	P value
Second-line regimen	AZT/XTC/LPV/r	469/630 (74·4)	1	-	1	-
	AZT/XTC/DTG	170/196 (86·7)	1·17 (1·05-1·31)	0·0038	1·18 (1·06-1·31)	0·0028
	TDF/XTC/DTG	200/267 (74·9)	1·00 (0·91-1·10)	0·96	1·00 (0·91-1·09)	0·98
Age at baseline	15-24	60/81 (74·1)	1	-	1	-
	25-34	296/397 (74·6)	1·01 (0·88-1·16)	0·89	1·01 (0·88-1·16)	0·89
	35-44	334/427 (78·2)	1·06 (0·92-1·21)	0·43	1·06 (0·93-1·21)	0·36
	45+	149/188 (79·3)	1·08 (0·94-1·23)	0·28	1·07 (0·94-1·22)	0·29
Gender	Male	325/432 (75·2)	1	-	1	-
	Female	514/661 (77·8)	1·03 (0·97-1·11)	0·33	1·05 (0·98-1·12)	0·13
Known tuberculosis at baseline	No	828/1075 (77·0)	1	-	1	-
	Yes	11/18 (61·1)	0·79 (0·55-1·13)	0·19	0·79 (0·56-1·13)	0·19
Recent viral load (copies/ml) at baseline	1,000 to <10,000	353/440 (80·2)	1	-	1	-
	10,000+	486/653 (74·4)	0·93 (0·87-0·99)	0·022	0·93 (0·87-1·00)	0·036
Recent CD4 count (cells/µl) at baseline	≤ 200	303/396 (76·5)	1	-	-	-
	201–350	203/269 (75·5)	0·99 (0·90-1·08)	0·78	-	-
	351–500	115/157 (73·2)	0·96 (0·86-1·07)	0·43	-	-
	> 500	100/126 (79·4)	1·04 (0·93-1·16)	0·51	-	-
	Missing	118/145 (81·4)	1·08 (0·99-1·17)	0·0904	-	-
Years on ART at baseline	< 2 year	302/405 (74·6)	1	-	-	-
	≥ 2 years	537/688 (78·1)	1·05 (0·97-1·13)	0·25	-	-
Time-period of switching to second-line treatment	Dec19-Feb20	157/199 (78·9)	1	-	-	-
	Mar20-May20	223/286 (78·0)	0·99 (0·91-1·06)	0·71	-	-
	Jun20-Aug20	253/335 (75·5)	0·95 (0·87-1·05)	0·33	-	-
	Sep20-Nov20	206/273 (75·5)	0·95 (0·86-1·06)	0·35		

Data are n/N (%), unless otherwise stated. [a]The primary exposure effect (retention-in-care at 12 months) is adjusted for all other variables in the table as potential confounders except CD4 count and Years on ART at baseline. ART = Antiretroviral treatment, AZT = Zidovudine, DTG = Dolutegravir, EFV = Efavirenz, LVP/r = Lopinavir-ritonavir, µl = Microliter, ml = Milliliter, NVP = Nevirapine, PLHIV = People living with HIV, RR = Risk ratio, TDF = Tenofovir disoproxil fumarate, XTC = Emtricitabine or Lamivudine.

**Table S 6. Univariable and multivariable Poisson regression models of factors associated with viral suppression (<50 copies/ml) at 12 months excluding 121 participants who changed their originally prescribed second-line regimen within 12 months after baseline (N = 713)**						
Variable	Level	Viral load at 12 months < 50 copies/ml n/N (%)	Unadjusted RR (95% CI)	P value	Adjusted RR[a] (95% CI)	P value
Second-line regimen	AZT/XTC/LPV/r	188/407 (46·2)	1	-	1	-
	AZT/XTC/DTG	84/137 (61·3)	1·27 (1·04-1·56)	0·019	1·29 (1·06-1·57)	0·0103
	TDF/XTC/DTG	103/169 (60·9)	1·32 (1·14-1·53)	<0·0001	1·31 (1·13-1·52)	<0·0001
Age at baseline	15-24	19/51 (37·3)	1	-	1	-
	25-34	140/257 (54·5)	1·47 (0·98-2·22)	0·065	1·50 (1·00-2·27)	0·053
	35-44	142/275 (51·6)	1·38 (0·90-2·12)	0·14	1·48 (0·97-2·27)	0·071
	45+	74/130 (56·9)	1·58 (1·04-2·40)	0·031	1·63 (1·07-2·49)	0·023
Gender	Male	132/271 (48·7)	1	-	1	-
	Female	243/442 (55·0)	1·15 (1·00-1·32)	0·042	1·17 (1·03-1·33)	0·016
Known tuberculosis at baseline	No	373/703 (53·1)	1	-	1	-
	Yes	2/10 (20·0)	0·40 (0·12-1·31)	0·13	0·41 (0·12-1·32)	0·14
Recent viral load (copies/ml) at baseline	1,000 to <10,000	177/301 (58·8)	1	-	1	-
	10,000+	198/412 (48·1)	0·84 (0·74-0·94)	0·0031	0·87 (0·78-0·98)	0·019
Recent CD4 count (cells/µl) at baseline	≤ 200	139/264 (52·7)	1	-	-	-
	201–350	79/167 (47·3)	0·91 (0·77-1·08)	0·27	-	-
	351–500	60/96 (62·5)	1·17 (0·98-1·40)	0·091	-	-
	> 500	41/86 (47·7)	0·93 (0·68-1·27)	0·64	-	-
	Missing	56/100 (56·0)	1·07 (0·84-1·36)	0·57	-	-
Years on ART at baseline	< 2 year	139/254 (54·7)	1	-	-	-
	≥ 2 years	236/459 (51·4)	0·94 (0·80-1·10)	0·43	-	-
Time-period of switching to second-line treatment	Dec19-Feb20	72/140 (51·4)	-	-	-	-
	Mar20-May20	97/189 (51·3)	1·01 (0·84-1·21)	0·89	-	-
	Jun20-Aug20	114/220 (51·8)	1·03 (0·86-1·22)	0·78	-	-
	Sep20-Nov20	92/164 (56·1)	1·08 (0·89-1·30)	0·45		
Data are n/N (%), unless otherwise stated. [a]The primary exposure effect (viral suppression at 12 months) is adjusted for all other variables in the table as potential confounders except CD4 count and Years on ART at baseline. ART = Antiretroviral treatment, AZT = Zidovudine, DTG = Dolutegravir, EFV = Efavirenz, LVP/r = Lopinavir-ritonavir, µl = Microliter, ml = Milliliter, NVP = Nevirapine, PLHIV = People living with HIV, RR = Risk ratio, TDF = Tenofovir disoproxil fumarate, XTC = Emtricitabine or Lamivudine.						

**Table S 7. Univariable and multivariable Poisson regression models of factors associated with viral suppression (<1000 copies/ml) at 12 months excluding 121 participants who changed their originally prescribed second-line regimen within 12 months after baseline (N = 713)**						
Variable	Level	Viral load at 12 months < 1000 copies/ml n/N (%)	Unadjusted RR (95% CI)	P value	Adjusted RR[a] (95% CI)	P value
Second-line regimen	AZT/XTC/LPV/r	284/407 (69·8)	1	-	1	-
	AZT/XTC/DTG	119/137 (86·9)	1·19 (1·07-1·33)	0·0017	1·20 (1·08-1·33)	0·00051
	TDF/XTC/DTG	133/169 (78·7)	1·13 (1·02-1·25)	0·020	1·11 (1·00-1·23)	0·046
Age at baseline	15-24	29/51 (56·9)	1	-	1	-
	25-34	189/257 (73·5)	1·28 (0·98-1·65)	0·066	1·31 (1·01-1·70)	0·043
	35-44	216/275 (78·5)	1·34 (1·02-1·77)	0·035	1·37 (1·04-1·81)	0·023
	45+	102/130 (78·5)	1·39 (1·06-1·83)	0·018	1·35 (1·02-1·79)	0·039
Gender	Male	212/271 (78·2)	1	-	1	-
	Female	324/442 (73·3)	0·95 (0·87-1·03)	0·23	0·95 (0·87-1·03)	0·22
Known tuberculosis at baseline	No	531/703 (75·5)	1	-	1	-
	Yes	5/10 (50·0)	0·65 (0·37-1·14)	0·13	0·66 (0·38-1·16)	0·15
Recent viral load (copies/ml) at baseline	1,000 to <10,000	250/301 (83·1)	1	-	1	-
	10,000+	286/412 (69·4)	0·85 (0·79-0·92)	<0·0001	0·84 (0·78-0·91)	<0·0001
Recent CD4 count (cells/µl) at baseline	≤ 200	201/264 (76·1)	1	-	-	-
	201–350	121/167 (72·5)	0·96 (0·87-1·06)	0·41	-	-
	351–500	79/96 (82·3)	1·08 (0·97-1·21)	0·16	-	-
	> 500	59/86 (68·6)	0·92 (0·78-1·09)	0·33	-	-
	Missing	76/100 (76·0)	1·01 (0·88-1·17)	0·84	-	-
Years on ART at baseline	< 2 year	198/254 (78·0)	1	-	-	-
	≥ 2 years	338/459 (73·6)	0·95 (0·88-1·01)	0·12	-	-
Time-period of switching to second-line treatment	Dec19-Feb20	111/140 (79·3)	-	-	-	-
	Mar20-May20	142/189 (75·1)	0·96 (0·85-1·08)	0·48	-	-
	Jun20-Aug20	154/220 (70·0)	0·91 (0·80-1·02)	0·098	-	-
	Sep20-Nov20	129/164 (78·7)	0·99 (0·88-1·10)	0·81		

Data are n/N (%), unless otherwise stated. [a]The primary exposure effect (viral suppression at 12 months) is adjusted for all other variables in the table as potential confounders except CD4 count and Years on ART at baseline. ART = Antiretroviral treatment, AZT = Zidovudine, DTG = Dolutegravir, EFV = Efavirenz, LVP/r = Lopinavir-ritonavir, µl = Microliter, ml = Milliliter, NVP = Nevirapine, PLHIV = People living with HIV, RR = Risk ratio, TDF = Tenofovir disoproxil fumarate, XTC = Emtricitabine or Lamivudine.
