# Supplementary material for: Clinical outcomes with second-line dolutegravir in people with virological failure on first-line non-nucleoside reverse transcriptase inhibitor-based regimens in South Africa: a retrospective cohort study
Source: Lancet Glob Health. 2023 Dec 21;12(2):e282–91. doi: 10.1016/S2214-109X(23)00516-8 (PMC10805003; doi:10.1016/S2214-109X(23)00516-8)
Supplement: Equitable Partnership Declaration [file mmc3.pdf]

THE LANCET
Global Health

Supplementary appendix 3

This Equitable Partnership Declaration (EPD) was submitted by the authors, and we reproduce it as supplied. It has not been peer reviewed. *The Lancet*'s editorial processes have not been applied to the EPD.

Supplement to: Asare K, Sookrajh Y, van der Molen J, et al. Clinical outcomes with second-line dolutegravir in people with virological failure on first-line non-nucleoside reverse transcriptase inhibitor-based regimens in South Africa: a retrospective cohort study. *Lancet Glob Health* 2023; published online Dec 21. https://doi.org/10.1016/S2214-109X(23)00516-8.

Equitable Partnership Declaration questions

Researcher considerations

1. Please detail the involvement that researchers who are based in the region(s) of study had during a) study design; b) clinical study processes, such as processing blood samples, prescribing medication, or patient recruitment; c) data interpretation; and d) manuscript preparation, commenting on all aspects. If they were not involved in any of these aspects, please explain why.

   This question is intended for international partnerships; if all your authors are based in the area of study, this question is not applicable.

   This should include a thorough description of their leadership role(s) in the study. Are local researchers named in the author list or the acknowledgements, or are they not mentioned at all (and, if not, why)? Please also describe the involvement of early career researchers based in the location of the study. Some of this information might be repeated from the Contributors section in the manuscript. Note: we adhere to ICMJE authorship criteria when deciding who should be named on a paper.

a) Study design:

All authors are currently based in South Africa, except JD.
KA is South African based working as an epidemiologist, PhD student and early career researcher at the Centre for the AIDS Programme of Research in South Africa (CAPRISA).
YS is a South African working as a senior medical practitioner at the eThekwini Municipality Health Unit.
JvM is South African based working as a data scientist at CAPRISA.
TK is a South African Senior Manager of Information at the eThekwini Municipality Health Unit.
LL is a South African working as a statistician, PhD student and early career researcher at CAPRISA.
RJL is a South African based senior clinical researcher and medical doctor at CAPRISA and KRISP.
KN is a South African senior clinical researcher and medical doctor and Deputy Director of CAPRISA.
PS is a South African senior medical practitioner at the eThekwini Municipality Health Unit.
RvH is a South African and Head of Health at the eThekwini Municipality Health Unit.
NG is a South African based senior clinical researcher and medical doctor and Head of Vaccines and Pathogenesis at CAPRISA.
JD was previously based in South Africa and holds an Associate Scientist position at CAPRISA alongside an Academic Clinical Lectureship at the University of Oxford

This manuscript is conducted as part of the "Strengthening Health Systems Through Audit and Programmatic Data Evaluation (SHAPE)" project. SHAPE is a collaboration between the Centre for the AIDS Programme of Research in South Africa (CAPRISA), the eThekwini Municipality Health Unit and the University of Oxford, aimed at using routinely collected ART programmatic data to answer locally relevant questions to improve HIV care. JD, NG and LL in their role at CAPRISA and YS, TK, PS and RvH in their roles at the eThekwini Municipality Health Unit were part of the initial stakeholder meetings and activities to conceptualise the SHAPE project, protocol development, obtaining ethical approval, and defining the research question for this specific analysis. KA and JvM are part of the SHAPE team at CAPRISA involved in activities including weekly meetings to discuss the design and execution of tasks related to the publication of this manuscript and other SHAPE project deliverables.

**b) Clinical study processes:**<br>YS, TK, PS and RvH in their roles at the eThekwini Municipality Health Unit lead HIV care provision and data capture and data management from health records into TIER.Net electronic database.
**c) Data interpretation:**<br>All authors were involved in data interpretation. Preliminary data is routinely shared with stakeholders of the municipality for discussion, feedback and to decide the worthiness of publication.
**d) Manuscript preparation:**<br>KA took the lead with the formal analysis and manuscript preparation with validation and support from JvM, LL, NG and JD. YS, TH, PS, RLJ, KN, RvH reviewed and provided feedback that significantly improved the manuscript's methodology, interpretation, and presentation.

2. Were the data used in your study collected by authors named on the paper, or have they been extracted from a source such as a national survey? ie, is this a secondary analysis of data that were not collected by the authors of this paper. If the authors of this paper were not involved in data collection, how were data interpreted with sufficient contextual knowledge?

   The Lancet Global Health *believe contextual understanding is crucial for informed data analysis and interpretation.*

Data used were extracted from TIER.Net electronic database used in South Africa to record demographics, clinical status, regimen, and clinic visit information of people receiving ART in public sector healthcare clinics. YS, PS and RvH lead the provision of clinical care, and TK leads the management of TIER.net data, from the clinics in this study and so provided local context. RJL, KN, NG and JD are HIV/TB clinicians who have worked in South Africa and also provided local and national context.

3. How was funding used to remunerate and enhance the skills of researchers and institutions based in the area(s) of study? And how was funding used to improve research infrastructure in the area of study?

   Potentially effective investments into long-term skills and opportunities within institutions could include training or mentorship in analytical techniques and manuscript writing, opportunities to lead

all or specific aspects of the study, financial remuneration rather than requiring volunteers, and other professional development and educational opportunities.

Improvements to research infrastructure could be funding of extended trial designs (such as platform trials) and use of master protocols to enable these designs, establishment of long-term contracts for research staff, building research facilities, and local control of funding allocation.

**Skills:** Funding for SHAPE has been used to fund KA, JvdM, LL and NG's work on this project. KA and LL are currently undertaking PhDs in Epidemiology and Biostatistics. SHAPE funding has been used to provide training for CAPRISA Research Fellows in data analysis and statistics using R.
**Research infrastructure:**<br>SHAPE funding is directly to CAPRISA who manage the grant. SHAPE funding has also been used to develop big data management capabilities and processes at CAPRISA, and to build long term collaborations with eThekwini Municipality and other KwaZulu-Natal Districts to allow improved monitoring and evaluation of the HIV/TB programme.

4. How did you safeguard the researchers who implemented the study?

Please describe how you guaranteed safe working conditions for study staff, including provision of appropriate personal protective equipment, protection from violence, and prevention of overworking.

All study staff worked under their respective institutional labour policies in accordance with the labour laws in South Africa. These laws outline employee rights regarding working hours, leave, and occupational safety.

<u>*Benefits to the communities and regions of study*</u>

5. How does the study address the research and policy priorities of its location?

How were the local priorities determined and then used to inform the research question? Who decided which priorities to take forward? Which elements of the study address those priorities?

The KwaZulu-Natal province of South Africa has the highest burden of HIV. In the KwaZulu-Natal province, the eThekwini Municipality records the highest district-level burden. Improved HIV care outcomes remains a top priority to ensure better quality of life in persons living with HIV while reducing transmission risk. Dolutegravir has been implemented for second-line antiretroviral therapy and this research from routine care confirms the worthiness of the policy change which was based on trial evidence. It also reveals interim bottlenecks for better outcomes during second-line treatment that can inform improved implementation for better outcomes.

6. How will research products be shared in the community of study?

For instance, will you be providing written or oral layperson summaries for non-academic information sharing? Will study data be made available to institutions in the region(s) of study? The Lancet Global Health *encourages authors to translate the summary (abstract) into relevant languages after paper editing; do you intend to translate your summary?*

The SHAPE project has periodic stakeholder meetings with the eThekwini Municipality/district directorates of the study clinics and the National TB/HIV Information Systems Data Request Committee. At these stakeholder meetings findings, published or unpublished of all recent analyses are shared through presentations and discussions. All publications are also shared on CAPRISA's monthly online newsletters to advance dissemination of research findings to the wider community. Results will also be presented at the KwaZulu-Natal Department of Health Research Day, and have been presented at the South African HIV Drug Resistance Workshop 2023 in Cape Town. The abstract will be translated into isiZulu and the results will be presented to the CAPRISA Community Advisory Board.

7. How were individuals, communities, and environments protected from harm?

a) *How did you ensure that sensitive patient data was handled safely and respectfully? Was there any potential for stigma or discrimination against participants arising from any of the procedures or outcomes of the study?*

In accordance with research ethics regulations and Protection of Participant Information Act (POPIA) of South Africa, we had access to de-identified data from TIER.Net electronic database. Based on CAPRISA's research confidentiality standard operating procedure all study computers and laptops are password protected and encrypted. All study activities in relation to participant data was done following CAPRISA's SOPs regarding data access and use.

b) *Might any of the tests be experienced as invasive or culturally insensitive?*

Not Applicable.

c) *How did you determine that work was sensitive to traditions, restrictions, and considerations of all cultural and religious groups in the study population?*

Not Applicable.

d) *Were biowaste and radioactive waste disposed of in accordance with local laws?*

Not Applicable.

e) *Were any structures built that would have impacted members of the community or the environment (such as handwashing facilities in a public space)? If so, how did you ensure that you had appropriate community buy-in?*

Not Applicable.

f) *How might the study have impacted existing health-care resources (such as staff workloads, use of equipment that is typically employed elsewhere, or reallocation of public funds)?*

Not Applicable.

8. Finally, please provide the title (eg, Dr/Prof, Mr/Mrs/Ms/Mx), name, and email address of an author who can be contacted about this statement. This can be the corresponding author.

**Name:** Dr Jienchi Dorward<br>**Email:** jienchi.dorward@caprisa.org
